# Mapping load transfer from the plantar surface of the foot to the walls of the total contact cast (TCC)

**DOI:** 10.1186/1757-1146-5-S1-O12

**Published:** 2012-04-10

**Authors:** Lindy Begg, Patrick McLaughlin, Leon Manning, Axel Kalpen, Mauro Vicaretti, John Fletcher, Joshua Burns

**Affiliations:** 1 Foot Wound Clinic, Department of Surgery, Westmead Hospital, 2145, Australia; 2 School of Biomedical and Health Sciences, Faculty of Health, Engineering and Science, Victoria University, Melbourne 8001, Australia; 3 Institute of Sport, Exercise and Active Living, Victoria University, Melbourne, 8001, Australia; 4 Novel Biomechanics Lab, Munich, Germany; 5 Institute for Neuroscience and Muscle Research, The Children’s Hospital at Westmead/Paediatric Gait Analysis Service of New South Wales/Faculty of Health Sciences, The University of Sydney, NSW, 2145, Australia

## Background

The mechanism of offloading plantar pressure with a TCC is by redistributing weight-bearing load across the entire plantar surface of the foot and increasing the plantar surface contact area [1]. An additional mechanism is by transfer of load to the cast walls however these studies relied on *indirect* methods [2-4]. No previous research has *directly* measured the load on the cast wall.

The aim of this pilot study was to:

1. Systematically map pressure between the walls of the TCC and the lower limb to identify those areas of greatest pressure.

2. To directly measure load transfer from the plantar surface of the foot to the cast walls.

## Materials and methods

A TCC was applied to a 20 year old healthy female and a 32 year old female with a 17 year history of Diabetes Mellitus without complications. The TCC was bi-valved and a capacitance sensor insole (pedar^®^, novel Gmbh, Germany) was placed on to the plantar area of the TCC and another into the participant’s sport shoe. Pliance^®^ sensors were *also* placed along the lower leg (pliance^®^, novel Gmbh, Germany). Both sensors collected data simultaneously. After completion of all seven trials measuring each location of the cast wall, the cast wall of the TCC was cut down to create a shoe-cast.

## Results

The two highest pressure locations from the cast-wall pliance^®^ sensors were: posterior to the lateral malleolus and the extensor retinaculum. The average force per step for the resultant cast wall load was 159.2N for the participant with diabetes and 104.8N for the participant without diabetes. With the use of direct measurement it was established that there was a load transfer of 34% from the plantar surface of the foot to the cast walls of a TCC worn by a participant with Diabetes and for the healthy participant 23%.

## Conclusions

The results supported the estimated values of 30-35% of load transfer by previous researchers who calculated cast wall data from *indirect* (plantar) measures.
